# Spatial and Temporal Variations in the Trophic Structure of Fish Assemblages in the Eastern Region of the Yellow Sea Determined by C- and N-Stable Isotope Ratios

**DOI:** 10.3390/biology14111521

**Published:** 2025-10-30

**Authors:** Dong-Young Lee, Jae Mook Jeong, Dongyoung Kim, Donghoon Shin, Chung Il Lee, Jeong-Hoon Lee, Hawsun Sohn, Hyun Je Park

**Affiliations:** 1 Department of Marine Ecology and Environment, Gangneung-Wonju National University, Gangneung 25457, Republic of Korea; 2 Fisheries Resources Research Center, National Institute of Fisheries Science, Tongyeong 53064, Republic of Korea

**Keywords:** fish community, food webs, stable isotopes, isotopic niches, Yellow Sea, fishery management

## Abstract

**Simple Summary:**

This study examined spatial and seasonal variations in the trophic structure of fish assemblages in the eastern Yellow Sea using carbon and nitrogen stable isotopes. Significant differences in δ^13^C and δ^15^N values were found among sites and seasons, though no consistent trend emerged. Phytoplankton-derived organic matter was identified as the main energy source for the regional food web. These findings improve understanding of how environmental changes influence marine trophic dynamics and support ecosystem-based fisheries management in the Yellow Sea.

**Abstract:**

This study aimed to determine how spatial and seasonal variations influence the trophic structure of fish assemblages in the eastern Yellow Sea during 2023. Carbon and nitrogen stable isotope analyses revealed significant but spatially variable differences in δ^13^C and δ^15^N values among seasons and sites, without consistent overarching trends. These results suggest that phytoplankton-derived organic matter predominantly supports the regional food web, with isotopic niche shifts reflecting local differences in prey availability and hydrographic conditions. The findings contribute to the development of adaptive, ecosystem-based fishery management strategies amid increasing anthropogenic and climatic pressures.

## 1. Introduction

The Yellow Sea is a marginal sea surrounded by the Korean Peninsula and China. It is semi-enclosed and serves as a critical junction connecting the East China Sea and the Bohai Sea [1]. The sea is relatively shallow, with a depth of less than approximately 70 m, and rich sediment deposits have formed extensive mudflats and tidal zones, fostering unique coastal landscapes [2]. Various physical processes, such as climatological features, freshwater input, tides, circulation, and currents, occur in the Yellow Sea and are known to cause highly variable environmental conditions [3,4]. The Yellow Sea is a biodiversity hotspot with high biological productivity worldwide, supporting productive fisheries essential for local economies and food security [5,6]. In particular, the eastern region of the Yellow Sea, also known as the West Sea of Korea, is of significant ecological and economic importance, characterized by its diverse fishery resources. The characteristics of this region as a significant fishery ground are likely driven by unique hydrographic conditions, nutrient influx, and broad tidal flats [7,8,9]. However, various human activities, including overfishing, pollution, and climate change, have significantly impacted regional fishery resources [8,10,11]. These anthropogenic effects have led to substantial depletion in the abundance of commercially important fish populations and the total biomass of fishery resources [11]. Pollution in this region is primarily associated with nutrient enrichment, organic effluent from aquaculture, and trace metal contamination from industrial sources. Previous studies have reported shifts in fish community structures and trophic dynamics in response to temperature anomalies, eutrophication, and nutrient loading in the Yellow Sea [6,12,13]. However, the spatio-temporal dynamics of trophic interactions remain poorly quantified in this region.

Because fish generally play an ecological role as integral components, changes in their distribution and abundance can alter ecological functions in the entire ecosystem [14]. In particular, the shift in dominant fish populations due to environmental change and/or anthropogenic impacts can have significant effects on trophic interactions between prey and predators, and thereby, food web structure, through trophic cascading in marine ecosystems [15,16]. Basic information on the food web structure of fish assemblages and their trophic responses to various anthropogenic and environmental factors must be obtained to understand the structure and function of ecosystems. Furthermore, scientific data on complex trophic networks can be used to ensure that ecosystem-based fishery management conserves resources [17,18].

Stable isotope analysis has been used as an effective chemical tracer to study prey-predator interactions and food webs in marine ecosystems [19,20]. In particular, stable carbon and nitrogen isotopes provide basic information about organic matter cycling and trophic structure within ecosystems through the isotopic signatures of prey items and consumers [21]. The usefulness of carbon- and nitrogen-stable isotopes is fundamentally based on the assumption that the isotope ratios in consumers reflect those in their foods with the predictable trophic fractionations of 0.3–1.3‰ for δ^13^C and 2–4‰ for δ^15^N [22,23,24]. This method for determining δ^13^C and δ^15^N values enables us to identify the origin of dietary sources for consumers, their trophic levels, and trophic pathways within a given ecosystem [19,25]. The application of stable isotopes has enabled us to describe the feeding strategies and trophic roles of fish populations and the trophic structure of fish assemblages in marine ecosystems [26,27,28,29].

In this study, we assessed spatial and temporal variations in the trophic structure of fish assemblages and their trophic levels in the eastern region of the Yellow Sea based on stable carbon and nitrogen isotope analyses. The aim of the study was to characterize the food web by determining the trophic interactions and trophic positions of fish assemblages within this ecosystem. We hypothesized that spatial and seasonal physical environmental differences would cause changes in the species composition and abundance of fish assemblages, thereby reflecting their trophic relationships and isotopic characteristics.

## 2. Materials and Methods

### 2.1. Study Sites

**Figure 1:**
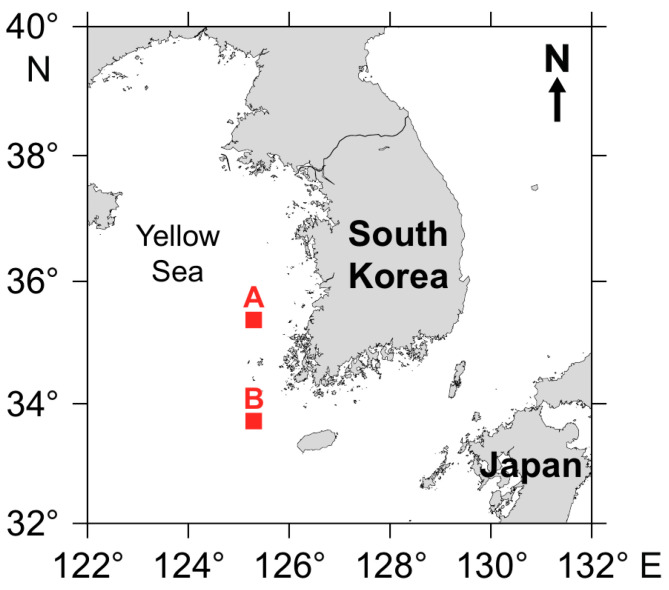
Map of the sampling areas in the eastern region of the Yellow Sea. The sampling sites (Site A, north and Site B, south) were located in the western coastal areas of the Korean peninsula.

Seasonal research cruises by a research vessel, R/V *Tamgu* 21, with support from the National Institute of Fisheries Science (NIFS) of Korea, were conducted at two sites (site A, 35°11′ N, 125°14′ E; site B, 33°46′ N, 125°6′ E) in the Yellow Sea (Figure 1). The average depth in the sampling area was 50.6 m and 72.4 m at St. A and St. B, respectively. The surface layer water temperatures in the study areas were relatively high in the summer (29.4 °C and 27.2 °C at St. A and B, respectively) compared to those in the winter (6.81 °C and 10.1 °C at St. A and B, respectively). Water temperatures in the bottom layer were relatively consistent compared to those in the surface layer in the summer (8.5 °C and 10.5 °C at St. A and B, respectively), compared to those in the winter (13.5 °C and 15.2 °C at St. A and B, respectively). The two stations were selected to represent distinct hydrographic and topographic environments of the eastern Yellow Sea, with St. A located on the shallow continental shelf and St. B in a deeper offshore area. These sites have been routinely monitored by the National Institute of Fisheries Science and are considered representative of the eastern Yellow Sea ecosystem. Seasonal sampling was conducted in February, May, August, and November 2023 to represent winter, spring, summer, and autumn conditions.

### 2.2. Sample Collection and Processing

All fish samples were collected using a trawl net (12 cm mesh in the main body, 8 cm mesh in the intermediate part, and 6 cm mesh in the cod end with a 2 cm cod end liner of the net) for over 30 min at 3 knots.

The fish samples collected on board were sorted and identified at the species level, with each individual length and weight measured to the nearest 0.1 cm and 0.1 g. Only the white muscle tissue of the fish was utilized for isotope analysis and was separated from the anterior dorsal regions using a knife, because it is metabolically stable and reflects long-term diet integration. Squid muscle tissue was carefully dissected from the anterior mantle. All animal samples were lyophilized, pulverized into a fine powder using a ball mill (MM200; Retsch GmbH, Haan, Germany), and kept frozen (−20 °C) until isotopic analysis.

The dominant zooplankton was collected through oblique towing using a Bongo net (2.0 m^2^ mouth opening, 500 μm mesh) equipped with a flowmeter. Zooplankton samples were preserved in 90% ethanol for taxonomic identification. The copepods and euphausiids were identified in the laboratory using a dissecting microscope.

Samples of suspended particulate organic matter (SPOM) were collected from the subsurface layers (approximately 20 L) on each sampling occasion. The water samples were pre-filtered through a 200 μm mesh sieve to remove zooplankton and large particles.d The pre-filtered water samples were re-filtered onto pre-combusted (450 °C for 4 h) Whatman GF/F glass fiber filters using a vacuum pump and stored at −20 °C until analysis. The SPOM samples for stable isotope analysis were fumed overnight with 1 N HCl to eliminate inorganic carbonates, oven-dried at 50 °C for 24 h, and then kept frozen at −20 °C until isotope analysis.

### 2.3. Stable Isotope Analyses

Powdered fish and zooplankton samples were weighed (0.5–1.0 mg) and transferred to tin combustion capsules. The filter samples for SPOM were enveloped in tin disks. All enveloped samples were combusted at 1020 °C in a CNSOH elemental analyzer (EA Isolink, Bremen, Germany), and the resultant gases were analyzed using a connected continuous-flow isotope ratio mass spectrometer (CF-IRMS; DELTA V PLUS, Bremen, Germany). The stable isotope ratios are presented in δ notation relative to conventional standard reference materials (Vienna Pee Dee Belemnite for carbon and atmospheric N_2_ for nitrogen), as follows: $\delta X\left(‰\right)= [\left(\frac{R_{sample}}{R_{standard}}\right)-\;1]\;\times \;10^{3}$, where X is ^13^C or ^15^N and R is ^13^C/^12^C or ^15^N/^14^N. The process was calibrated using the following reference standards: sucrose (ANU C_12_H_22_O_11_; NIST, Gaithersburg, MD, USA) for carbon and ammonium sulfate ([NH_4_]_2_SO_4_; NIST) for nitrogen. The analytical precision was obtained using 20 replicates of urea with 0.15‰ and 0.18‰ levels for δ^13^C and δ^15^N, respectively. The isotope analytical procedure followed established protocols for marine trophic studies [19,21].

Lipid correction was conducted because of biases in the δ^13^C values of fish species depending on the concentration of ^13^C-depleted lipids. Thus, following [30], we applied lipid correction to fish tissues above 3.5 of the mass C:N ratios, as these values may indicate a potential lipid bias. For C:N ratios greater than 3.5, lipid correction was conducted as follows: $\delta^{13}C_{normalized}=\delta^{13}C_{untreated}-3.32+0.99\times C:N(ratios)$, where $\delta^{13}C_{untreated}$ and $\delta^{13}C_{normalized}$ are the calculated and lipid-normalized values of the sample, respectively.

The TP of fish was measured using the equation: ${TP}_{i}={(\delta }^{15}N_{i}-\delta^{15}N_{baseline})/\Delta^{15}N+2$, where δ^15^N_i_ represents the mean δ^15^N of the fish species, δ^15^N_baseline_ is the mean δ^15^N of the food web baseline, $\Delta^{15}N$ is the enrichment factor with 3.4‰ [24] in δ^15^N per TP, and 2 represents the baseline TP. In this study, the trophic baseline was considered the δ^15^N value of the calanoid copepods at each sampling site.

### 2.4. Data Analyses

Statistical analyses were performed using SPSS software (ver. 21.0; IBM Corp., Armonk, NY, USA). Normality and homogeneity of variance were tested prior to statistical analyses for all datasets using Shapiro–Wilk and Levene’s tests, respectively. One-way analysis of variance (ANOVA), followed by Tukey’s honest significant difference (HSD) multiple comparison post hoc test, was conducted to identify significant differences in the δ^13^C and δ^15^N values of fish species among the sampling sites and seasons. Permutational multivariate analysis of variance (PERMANOVA) was performed to test for significant differences in the isotopic values of SPOM, zooplankton, and fish consumers among sampling sites and seasons using PRIMER version 6 (PRIMER-e, Auckland, New Zealand) with the PERMANOVA + PRIMER add-on [31].

Isotopic niche parameters (total area, TA; and sample size-corrected standard ellipse area, SEAc) of fish consumers were used to test spatial and seasonal differences in the trophic structure using the Stable Isotope Bayesian Ellipses in the R (SIBER) package within R software. The TA values were estimated using a convex hull area formed by the points of all species in a δ^13^C–δ^15^N biplot, representing the total amount of isotopic niche space for consumers [32]. SEAc values were quantified using the isotopic niche space to overcome problems caused by small sample sizes [33].

## 3. Results

### 3.1. Composition of Fish Assemblages

The species number and abundance (individuals/km^2^) of the fish assemblages varied seasonally, ranging from 11 (St. A in February) to 36 (St. A in November) and from 3881 (St. B in November) to 47,159 (St. A in November), respectively (Table 1). Univariate ecological indices of richness (*R*), evenness (*J*), and diversity (*H*′) widely varied among seasons, ranging from 1.03 (St. A in February) to 3.16 (St. A in November), 0.40 (St. B in May) to 0.86 (St. B in November), and 1.24 (St. B in May) to 2.64 (St. B in November), respectively.

### 3.2. Stable Isotope Values of SPOM and Zooplankton

The δ^13^C and δ^15^N values of SPOM differed significantly among the four seasons (PERMANOVA, pseudo-*F*_3, 37_ = 18.99, *p* = 0.001) and between the two sites (pseudo-*F*_1, 37_ = 20.77, *p* = 0.001), and a significant effect of the interaction term (season × site; pseudo-*F*_3, 37_ = 6.47, *p* = 0.001) was observed (Table 2). The isotopic values ranged from −22.9 ± 0.1‰ (St. A in February) to −19.6 ± 1.0‰ (St. B in February) and from 4.8 ± 0.3‰ (St. B in February) to 7.4 ± 0.7‰ (St. A in November), respectively.

Significant differences in the δ^13^C and δ^15^N values of both calanoid copepods and euphausiids were found among seasons (pseudo-*F*_3, 23_ = 9.51, *p* = 0.001; pseudo-*F*_3, 23_ = 5.90, *p* = 0.006, respectively) and between sites (pseudo-*F*_3, 23_ = 4.36, *p* = 0.024; pseudo-*F*_3, 23_ = 9.03, *p* = 0.001, respectively) (Table 2). There were no significant interaction term effects in either case (season × site; pseudo-*F*_3, 23_ = 0.38, *p* = 0.822 for copepods; pseudo-*F*_3, 23_ = 0.62, *p* = 0.654 for euphausiids). The overall δ^13^C and δ^15^N values ranged from –22.9 ± 0.5‰ (St. A in November) to −20.9 ± 0.8‰ (St. B in February) and from 5.7 ± 0.8‰ (St. B in February) to 7.5 ± 0.3‰ (St. B in May) for copepods, and from −22.3 ± 0.5‰ (St. A in November) to −20.8 ± 0.8‰ (St. B in February) and from 6.4 ± 0.9‰ (St. B in February) to 7.9 ± 0.3‰ (St. A in August) for euphausiids, respectively.

### 3.3. Stable Isotope Values of Fish Assemblages

**Figure 2:**
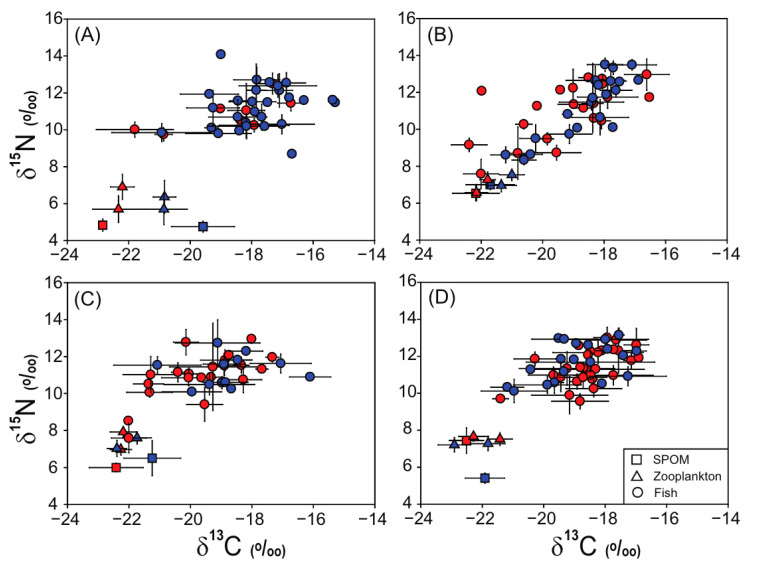
Dual isotope plots of δ^13^C and δ^15^N values of basal resources, zooplankton, and fish assemblages collected in the eastern regions of the Yellow Sea (site A and site B) during February (**A**), May (**B**), August (**C**), and November (**D**) 2023. Values are presented as mean δ^13^C and δ^15^N (‰ ± 1 SD).

Isotopic values of fish assemblages differed significantly among seasons (pseudo-*F*_3, 447_ = 3.73, *p* = 0.007) and between sites (pseudo-*F*_1, 447_ = 3.64, *p* = 0.047), whereas no significant effect of the interaction term of season × site (pseudo-*F*_3, 447_ = 1.43, *p* = 0.226) was found (Figure 2 and Table 3). Individually, there was a significant difference in the δ^13^C values of fish consumers among seasons (ANOVA, *F*_3, 447_ = 6.48, *p* < 0.001) and between sites (ANOVA, *F*_1, 447_ = 6.82, *p* = 0.009); the overall mean values ranged from −19.6 ± 1.3‰ (St. A in August) to −18.0 ± 1.3‰ (St. B in February) (Tables S1–S4). In contrast, no significant difference in their δ^15^N values was found among seasons (ANOVA, *F*_3, 447_ = 1.20, *p* = 0.311) or between sites (ANOVA, *F*_1, 447_ = 1.58, *p* = 0.209), which ranged from 11.0 ± 1.1‰ (St. A in August) to 11.5 ± 1.1‰ (St. B in November).

### 3.4. Trophic Positions and Isotopic Niches of Fish Assemblages

**Figure 3:**
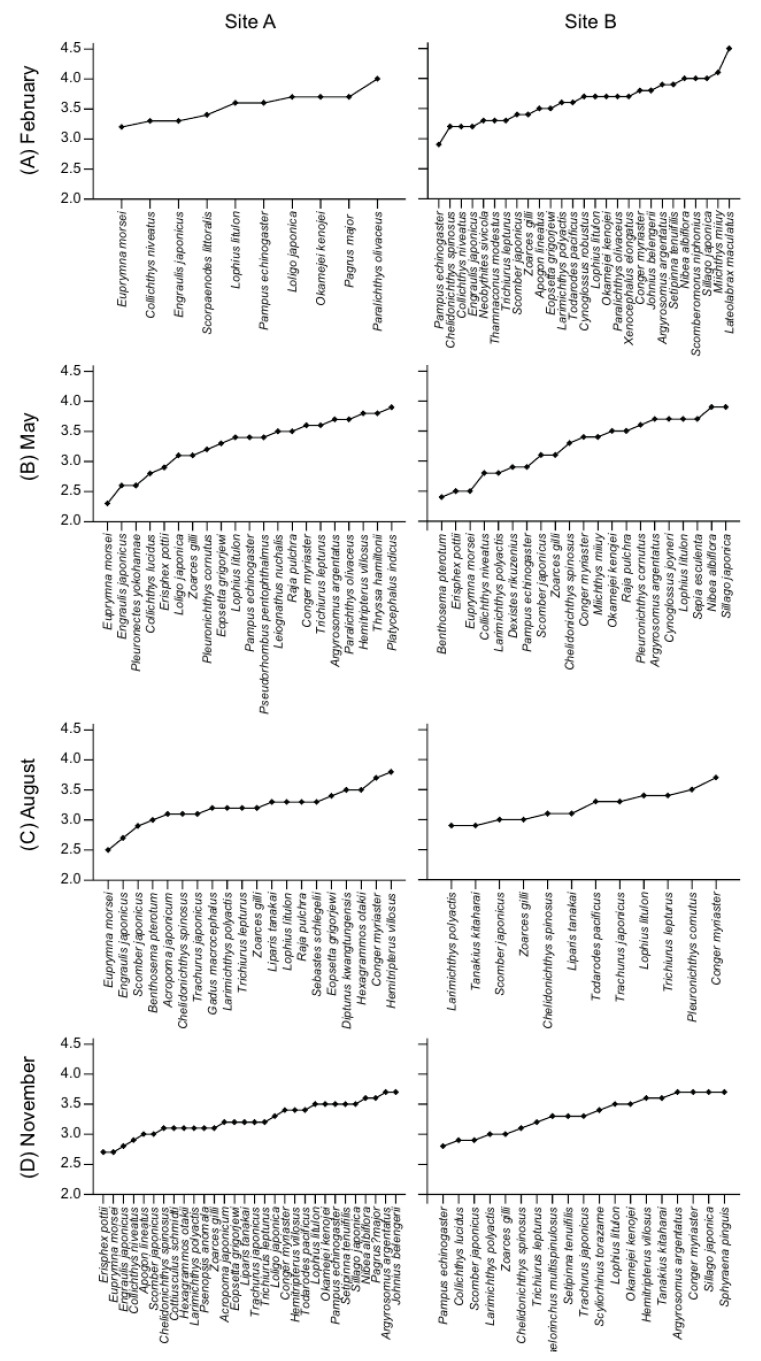
The ranges of trophic positions (TP) of fish assemblages collected in the eastern regions of the Yellow Sea (site A and site B) during February (**A**), May (**B**), August (**C**), and November (**D**) 2023.

The TP values of the fish assemblages differed significantly among seasons (ANOVA, *F*_3, 161_ = 6.56, *p* = 0.001), whereas no significant difference was found between sites (ANOVA, *F*_1, 161_ = 0.34, *p* = 0.562). The mean TP values ranged from 3.22 ± 0.30 (St. A in August) to 3.64 ± 0.34 (St. B in February) (Figure 3).

**Figure 4:**
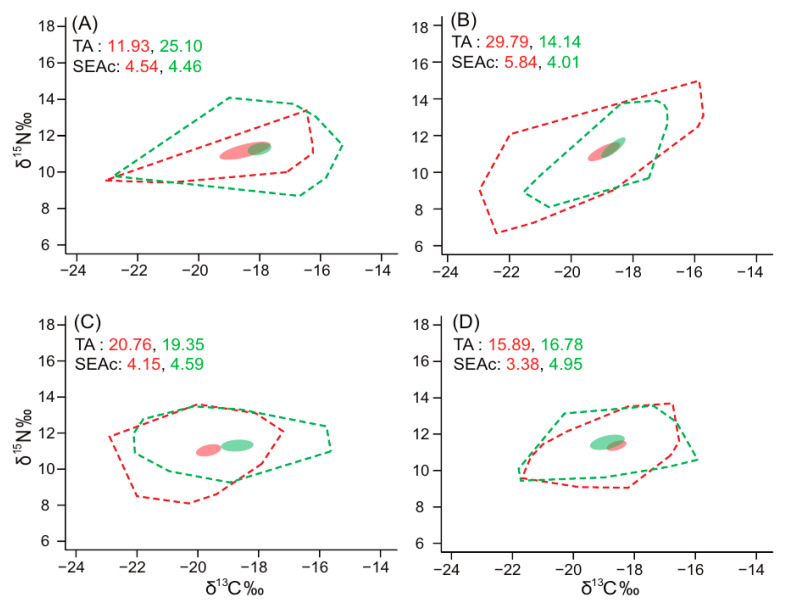
The isotopic niche areas (‰^2^) of fish assemblages collected in the eastern regions of the Yellow Sea (site A, red and site B, green) during February (**A**), May (**B**), August (**C**), and November (**D**) 2023 estimated as total area (TA, dotted line) and standard ellipse area (SEAc, solid line).

The isotopic niches (‰^2^) of fish assemblages estimated by the TA and SEAc values showed different seasonal patterns between the sites (Figure 4). The TA and SEAc values ranged from 11.93 (February) and 3.38 (November) to 29.79 (May) and 5.84 (May) for St. A and from 14.14 (May) and 4.01 (May) to 25.10 (February) and 4.95 (November) for St. B, respectively.

## 4. Discussion

Our study presents spatial and seasonal variations in the trophic structure of fish communities in the eastern region of the Yellow Sea by analyzing their carbon- and nitrogen-stable isotope ratios during four seasons. The main results indicated that differences in the species composition and abundance of fish communities in the sampling areas occurred with spatial and seasonal variations in their trophic structures, with distinct isotopic niche indices. These findings are consistent with those of several previous studies that showed that spatial and temporal variations in the abundance and composition of fish assemblages related to oceanographic environmental changes have many influences on their trophic relationships, and, thereby, on the food web structure [34,35,36]. Because food webs are complex ecological networks connected by many trophic relationships under various environmental conditions, our study provides information on the trophic responses of fish communities to environmental changes and/or anthropogenic effects in the Yellow Sea.

In our study, the species diversity of fish communities in the sampling areas (11–36 at St. A and 14–30 at St. B) was similar to and/or relatively higher than that previously reported for the Yellow Sea [36,37,38]. There was no clear seasonal trend in the species diversity of fish assemblages at either site. Furthermore, despite the spatial differences in community indices, verifying the spatial trends in the species composition and abundance of fish assemblages related to specific environmental conditions between the two sites was challenging. In general, the dynamics of species composition and abundance in fish communities in temperate regions are well connected to the consequences of seasonal shifts in oceanographic environmental conditions [39,40,41]. Seasonal variations in major environmental factors (e.g., water temperature and chlorophyll *a*) can affect the species abundance and composition of fish assemblages by changing their feeding, growth, reproduction, distribution, and movement [42,43]. Seawater temperature may be the most important factor controlling spatial and temporal variations in the species composition and abundance of fish communities in the Yellow Sea [44,45]. Thus, our results suggested that seasonal and spatial variations in the species composition and abundance of fish assemblages may be key features of fish communities in the coastal waters of the Yellow Sea, which makes identifying patterns associated with oceanographic environmental changes resulting from seasonal and regional differences challenging.

Our results revealed spatial and seasonal variations in the isotopic values of SPOM, which were consistent with previously reported ranges (−25 to −18‰ for δ^13^C and 2 to 10‰ for δ^15^N) of organic matter derived from phytoplankton in the Yellow Sea and the Korean coastal waters [36,46,47,48]. Spatial and seasonal variations in the δ^13^C and δ^15^N of SPOM commonly occur in coastal ecosystems and are strongly influenced by changes in physical, chemical, and biological factors under varying environmental conditions [48,49,50]. These results suggested that the organic matter derived from phytoplankton is a major source of SPOM in these areas. Moreover, the δ^13^C and δ^15^N values of the zooplankton groups revealed spatial and seasonal tendencies similar to those of the SPOM. Their δ^13^C and δ^15^N values also overlapped with previously reported ranges of general copepods (−24 to −21‰ for δ^13^C and 4 to 7‰ for δ^15^N) and euphausiids (−23 to −18‰ for δ^13^C and 4 to 8‰ for δ^15^N) in the Yellow Sea and the Korean coastal waters [29,51,52,53]. Overall, our results suggested that the spatial and seasonal isotopic ranges and variabilities of SPOM and zooplankton groups represent general patterns found in temperate coastal waters, including the Yellow Sea.

The fish assemblages in our study appeared to have significant spatial and seasonal differences in isotopic values despite slight overall shifts. However, in all cases, the distribution of δ^13^C and δ^15^N values for fish consumers on the dual isotope plots showed a general increasing pattern along their TPs, with isotopic enrichment per trophic level [35,54]. In addition, the trophic continuum of the δ^13^C and δ^15^N ranges of fish consumers linked from the points of SPOM and zooplankton groups suggested a major contribution of phytoplankton-derived organic matter to their nutrition. The feeding ecology of fish species is generally influenced by various factors such as food items, feeding zones, and trophic interactions [55]. The trophic status of fish consumers is well reflected in their isotopic distributions, leading to an understanding of nutritional resources, changes in fish diets, and even the trophic functioning of habitats [56]. Several studies have reported that benthic and benthopelagic fishes, defined by their inhabiting depth, generally have higher δ^13^C and δ^15^N values than pelagic fishes [35,57]. This tendency enabled us to classify the benthic and pelagic food webs of fish assemblages by tracing organic matter pathways. Similarly, in our study, typical pelagic fishes such as anchovies, squids, and mackerel also showed relatively low δ^13^C and δ^15^N values compared to benthic and benthopelagic fishes such as flounder, gurnards, and goosefish. However, it was difficult to distinguish between the benthic and pelagic isotopic pathways classified by their feeding patterns because of the overall narrow ranges of δ^13^C and δ^15^N in fish consumers in all seasons. Such isotopic ranges in fish consumers are likely related to the contribution of phytoplankton-derived organic matter as a major nutritional source in both benthic and pelagic food webs [36,58]. Furthermore, the low water depth (50–75 m) in the sampling areas, which increases the direct trophic connection of phytoplankton-derived organic matter through pelagic and benthic coupling, may also support this explanation [59]. Therefore, regardless of the season, the isotopic distributions of fish assemblages in the eastern Yellow Sea represent a general trophic continuum in coastal fish food webs supported by phytoplankton-derived organic matter.

Our study showed that microalgal sources significantly supported the trophic structure of fish assemblages. However, despite the importance of phytoplankton-derived organic matter in the sampling areas, spatial variability in the isotope signatures and species composition of fish communities was observed. The feeding habits of fish species generally exhibit trophic plasticity and/or opportunistic foraging performance concerning food availability [60,61]. The feeding behavior of fish consumers can be directly influenced by changing environmental conditions, resulting in spatial and seasonal variability in prey items and dynamics of relative resource contribution [62,63]. Such characteristics of individual fish species and their trophic interactions may lead to spatial and seasonal variability in the trophic structure of fish communities. Similarly, the community dynamics of fish assemblages are generally controlled by changing ambient environmental conditions. The distribution and migration of fish species may be closely related to oceanographic environmental conditions, including prey items, owing to their sensitive physiological responses, which can play an important role in the regulation of community structures [64,65]. Moreover, the dynamics of species composition and the diversity of fish assemblages as trophic components can have various effects on the organization of the food web structure. Ref. [36] reported that spatial and seasonal variabilities in the species composition of fish communities may affect their isotopic differences by altering the trophic interactions and pathways between the central and eastern regions of the Yellow Sea. Accordingly, the spatial differences in the trophic structures of fish communities between the sampling areas were primarily influenced by site-specific variability in the species composition of the community structure linked to different oceanographic conditions.

In our study, the spatial and seasonal variabilities in the trophic structure of fish assemblages were compressively estimated using isotopic niche parameters, such as TA and SEAc. In general, isotopic niche indices serve as indicators for evaluating the diversity of trophic pathways and spatiotemporal changes in trophic structure within aquatic ecosystems [32,66]. Our isotopic data demonstrated no clear spatial or seasonal trends in the isotopic niche indices of fish assemblages, owing to high variability. Several studies have reported that spatial and seasonal variations in the isotopic niches of fish assemblages are commonly attributed to changes in regional environmental conditions and food availability [53,67,68]. Furthermore, a shift in the community structure of fish assemblages (e.g., species abundance and composition) related to environmental factors can lead to changes in trophic relationships within the food web, which is represented by altering isotopic niche parameters such as TA and SEAc [35,69]. Actually, no spatial and seasonal trends in the community indices in our study, as mentioned above, may be reflected in the variability for the isotopic niche widths. Overall, such spatial and seasonal variabilities in the isotopic niche parameters were likely due to regional differences in environmental conditions and the changes in community indices of fish assemblages interacting with them. Zooplankton isotopic values showed strong coupling with SPOM and indicated a phytoplankton-based carbon source supporting higher trophic levels, consistent with the fish isotopic patterns observed. However, invertebrate biomass data were not available for quantitative modeling, which limits the full reconstruction of trophic fluxes. Future studies integrating compound-specific isotope analysis and longer time-series sampling would strengthen the interpretation of trophic connectivity.

## 5. Conclusions

This study revealed the spatial and seasonal variations in the trophic structure and community indices of fish assemblages in the eastern Yellow Sea. The isotopic distributions and niche indices of fish assemblages showed no clear spatial or seasonal trends, suggesting an effect of site-specific variability in environmental factors and community characteristics linked to different ambient environmental conditions. Specifically, such variability might result in extensive alteration of organic matter pathways and trophic relationships among fish consumers within the ecosystem. Our results implied that evaluating trophic status and food web structure based on the community compositions of fish assemblages and their stable isotope ratios likely reflects ecological changes in response to changing environmental conditions. Overall, this study is important for understanding the ecological role of fishery resources and enhancing ecologically based fishery management in the eastern region of the Yellow Sea under severe anthropogenic effects and climate change.

## Figures and Tables

**Table 1:** The species number and abundance (individuals/km^2^) and univariate ecological indices (richness, *R*; evenness, *J*; diversity, *H*′) of fish assemblages collected in the eastern regions of the Yellow Sea (site A and site B) in February (winter), May (spring), August (summer), and November (fall) 2023.

Species Name	February		May		August		November	
	St. A	St. B	St. A	St. B	St. A	St. B	St. A	St. B
Total species number	11	30	24	23	25	14	36	23
Total individuals	6107	13,193	12,855	22,871	8206	11,766	47,159	3881
Richness (*R*)	1.03	2.95	2.33	2.09	2.55	1.28	3.16	2.54
Evenness (*J*)	0.77	0.64	0.78	0.40	0.66	0.51	0.49	0.86
Diversity (*H*′)	1.77	2.95	2.33	2.09	2.55	1.28	3.16	2.54

**Table 2:** δ^13^C and δ^15^N values of organic matter (SPOM, suspended particulate organic matter) and zooplankton (calanoid copepods and euphausiids) collected in the eastern regions of the Yellow Sea (site A and site B) in February (winter), May (spring), August (summer), and November (fall) 2023. PERMANOVA test of δ^13^C and δ^15^N values for each potential food source among seasons and between sampling sites. Bold-face font indicates significance at *p* < 0.05. Data represent mean ± 1 SD.

		St. A					St. B			
Potential food source		δ^13^C		δ^15^N			δ^13^C		δ^15^N	
	n	Mean	SD	Mean	SD	n	Mean	SD	Mean	SD
February										
SPOM	4	−22.8	0.1	4.8	0.3	4	−19.6	1.0	4.8	0.3
Copepods	3	−22.3	0.8	5.7	0.7	3	−20.9	0.8	5.7	0.8
Euphausiids	3	−22.2	0.4	6.9	0.7	3	−20.8	0.4	6.4	0.9
May										
SPOM	5	−22.2	0.8	6.5	0.4	5	−21.7	0.8	7.0	0.3
Copepods	3	−21.8	0.3	7.3	0.4	3	−21.0	0.4	7.5	0.3
Euphausiids	3	−22.2	0.4	6.6	0.4	3	−21.3	0.5	7.0	0.3
August										
SPOM	5	−22.4	0.9	6.0	0.2	5	−21.2	0.9	6.5	0.9
Copepods	3	−22.3	0.3	7.0	0.3	3	−22.4	0.3	7.0	0.4
Euphausiids	3	−22.2	0.4	7.9	0.3	3	−21.7	0.5	7.6	0.3
November										
SPOM	5	−22.5	0.6	7.4	0.7	5	−21.9	0.6	5.4	0.3
Copepods	3	−22.9	0.5	7.2	0.4	3	−21.8	0.5	7.3	0.4
Euphausiids	3	−22.3	0.5	7.7	0.2	3	−21.4	0.4	7.5	0.3
PERMANOVA test	Season				Site				Interaction	
	pseudo-*F*		*p*		pseudo-*F*		*p*		pseudo-*F*	*p*
SPOM	18.99		**0.001**		20.76		**0.001**		6.47	**0.001**
Copepods	9.51		**0.001**		4.36		**0.024**		0.38	0.822
Euphausiids	5.90		**0.006**		9.03		**0.001**		0.62	0.654

**Table 3:** Mean δ^13^C, δ^15^N, and trophic positions (TP) of fish assemblages collected in the eastern regions of the Yellow Sea (site A and site B) in February (winter), May (spring), August (summer), and November (fall) 2023. PERMANOVA test of δ^13^C and δ^15^N values for fish assemblages among seasons and between sampling sites. Bold-face font indicates significance at *p* < 0.05. Data represent mean ± 1 SD.

		St. A						St. B					
		δ^13^C		δ^15^N		TP		δ^13^C		δ^15^N		TP	
	n	Mean	SD	Mean	SD	Mean	SD	Mean	SD	Mean	SD	Mean	SD
February	27	−18.5	1.8	11.2	1.1	3.53	0.26	−18.0	1.0	11.5	1.2	3.64	0.34
May	63	−18.9	1.7	11.1	1.7	3.30	0.45	−18.6	1.3	11.2	1.9	3.26	0.47
August	60	−19.6	1.3	11.1	1.1	3.22	0.30	−18.7	1.5	11.3	1.0	3.23	0.25
November	79	−18.6	1.2	11.4	1.0	3.24	0.27	−18.9	1.5	11.6	1.1	3.31	0.30
PERMANOVA test	Season				Site				Interaction				
	pseudo-*F*		*p*		pseudo-*F*		*p*		pseudo-*F*		*p*		
	3.73		**0.007**		3.64		**0.047**		1.43		0.226		

## Data Availability

The data presented in this study are available on request from the corresponding author.

## Supplementary Materials

The following supporting information can be downloaded at: https://www.mdpi.com/article/10.3390/biology14111521/s1, Table S1: δ^13^C, δ^15^N, and trophic positions (TP) of fish assemblages collected in the eastern regions of the Yellow Sea (site A and site B) in February 2023. Data represent mean ± 1 SD; Table S2: δ^13^C, δ^15^N, and trophic positions (TP) of fish assemblages collected in the eastern regions of the Yellow Sea (site A and site B) in May 2023. Data represent mean ± 1 SD; Table S3: δ^13^C, δ^15^N, and trophic positions (TP) of fish assemblages collected in the eastern regions of the Yellow Sea (site A and site B) in August 2023. Data represent mean ± 1 SD; Table S4: δ^13^C, δ^15^N, and trophic positions (TP) of fish assemblages collected in the eastern regions of the Yellow Sea (site A and site B) in November 2023. Data represent mean ± 1 SD.
