# Supplementary material for: Spatial and Temporal Variations in the Trophic Structure of Fish Assemblages in the Eastern Region of the Yellow Sea Determined by C- and N-Stable Isotope Ratios
Source: Biology (Basel). 2025 Oct 30;14(11):1521. doi: 10.3390/biology14111521 (PMC12650081; doi:10.3390/biology14111521)
Supplement: Supplementary file 1 [file biology-14-01521-s001.zip › biology-3954556-supplementary.pdf]

Supplementary Table S1 $\delta^{13}C$, $\delta^{15}N$, and trophic positions (TP) of fish assemblages collected in the eastern regions of the Yellow Sea (site A and site B) in February 2023. Data represent mean ± 1SD.

Species name	St. A						St. B					
	n	$\delta^{13}C$		$\delta^{15}N$		TP	n	$\delta^{13}C$		$\delta^{15}N$		TP
		Mean	S.D.	Mean	S.D.			Mean	S.D.	Mean	S.D.	
Apogon lineatus							3	–18.5	0.9	10.7	0.9	3.5
Argyrosomus argentatus							3	–17.1	0.4	12.1	0.9	3.9
Chelidonichthys spinosus							1	–18.4		9.9		3.2
Collichthys niveatus	3	–21.8	1.3	10.0	0.4	3.3	3	–20.9	1.6	9.8	0.5	3.2
Conger myriaster							3	–19.4	0.9	11.9	0.2	3.8
Cynoglossus robustus							1	–15.3		11.5		3.7
Engraulis japonicus	2	–17.9	1.1	10.2	0.4	3.3	2	–19.1	1.1	9.8	0.2	3.2
Eopsetta grigorjewi							4	–17.7	0.1	10.7	0.7	3.5
Euprymna morsei	3	–20.9	0.3	9.8	0.4	3.2						
Johnius belengerii							2	–16.8	0.1	11.7	0.2	3.8
Larimichthys polyactis							1	–17.9		11.0		3.6
Lateolabrax maculatus							1	–19.0		14.1		4.5
Loligo japonica	3	–18.4	0.3	11.6	0.0	3.7						
Lophius litulon	2	–19.0	0.1	11.1	0.1	3.6	7	–18.0	0.8	11.5	0.3	3.7
Miichthys miiuy							5	–17.8	0.7	12.7	0.7	4.1
Neobythites sivicola							3	–19.3	0.2	10.1	0.1	3.3
Nibea albiflora							3	–17.1	1.3	12.4	0.6	4.0
Okamejei kenojei	5	–16.7	0.4	11.4	0.4	3.7	1	–15.4		11.6		3.7
Pagrus major	1	–17.5		11.5		3.7						
Pampus echinogaster	2	–18.2	0.6	11.0	1.5	3.6	1	–16.7		8.7		2.9
Paralichthys olivaceus	6	–17.3	0.5	12.5	0.6	4.0	1	–16.3		11.6		3.7
Scomber japonicus							3	–17.0	1.1	10.3	0.5	3.4
Scomberomorus niphonius							1	–17.4		12.6		4.0
Scorpaenodes littoralis	1	–18.4		10.5		3.4						
Setipinna tenuifilis							2	–17.8	0.3	12.1	1.4	3.9
Sillago japonica							3	–16.9	0.6	12.5	0.5	4.0
Thamnaconus modestus							1	–17.6		10.2		3.3
Todarodes pacificus							3	–19.3	0.9	11.2	0.9	3.6
Trichiurus lepturus							3	–18.2	1.2	10.2	0.7	3.3
Xenocephalus elongatus							1	–18.4		11.3		3.7
Zoarces gilli							3	–18.2	0.1	10.4	0.3	3.4

Supplementary Table S2 δ^{13}C, δ^{15}N, and trophic positions (TP) of fish assemblages collected in the eastern regions of the Yellow Sea (site A and site B) in May 2023. Data represent mean ± 1SD.

Species name	St. A						St. B					
	n	δ^{13}C		δ^{15}N		TP	n	δ^{13}C		δ^{15}N		TP
		Mean	S.D.	Mean	S.D.			Mean	S.D.	Mean	S.D.	
Argyrosomus argentatus	3	–19.0	0.1	12.2	1.0	3.7	9	–17.8	0.5	12.6	0.8	3.7
Benthosema pterotum							3	–20.6	0.4	8.3	0.3	2.4
Chelidonichthys spinosus							3	–18.4	1.0	11.5	0.4	3.3
Collichthys lucidus	3	–22.4	0.6	9.2	0.4	2.8						
Collichthys niveatus							6	–20.2	0.5	9.5	0.7	2.8
Conger myriaster	1	–22.0		12.1		3.6	2	–18.4	0.2	11.7	1.9	3.4
Cynoglossus joyneri							1	–16.9		12.7		3.7
Dexistes rikuzenius							1	–18.9		10.1		2.9
Engraulis japonicus	3	–19.5	0.8	8.7	0.4	2.6						
Eopsetta grigorjewi	3	–18.7	0.3	11.2	0.1	3.3						
Erisphex pottii	3	–19.8	0.2	9.5	0.3	2.9	3	–20.4	0.4	8.6	0.1	2.5
Euprymna morsei	3	–22.0	0.6	7.6	0.8	2.3	2	–21.2	0.5	8.6	0.4	2.5
Hemitripterus villosus	1	–18.1		12.7		3.8						
Larimichthys polyactis							3	–19.1	0.7	9.7	0.5	2.8
Leiognathus nuchalis	1	–16.5		11.7		3.5						
Loligo japonica	3	–20.6	0.3	10.3	0.3	3.1						
Lophius litulon	3	–19.0	0.5	11.4	0.2	3.4	5	–18.3	0.9	12.6	0.8	3.7
Miichthys miiuy							5	–17.1	0.8	11.9	3.7	3.4
Nibea albiflora							2	–18.0	0.6	13.5	0.4	3.9
Okamejei kenojei							1	–17.9		11.9		3.5
Pampus echinogaster	2	–20.2	0.1	11.3	0.0	3.4	1	–17.7		10.1		2.9
Paralichthys olivaceus	6	–18.1	0.3	12.5	0.5	3.7						
Platycephalus indicus	8	–16.6	0.7	13.0	0.8	3.9						
Pleuronectes yokohamae	6	–20.8	0.4	8.7	1.4	2.6						
Pleuronichthys cornutus	1	–18.3		10.6		3.2	1	–18.2		12.4		3.6
Pseudorhombus pentophthalmus	3	–18.3	0.3	11.4	0.1	3.4						
Raja pulchra	6	–17.8	0.9	11.7	0.6	3.5	3	–17.6	0.5	12.1	0.4	3.5
Scomber japonicus							3	–18.1	0.9	10.6	1.0	3.1
Sepia esculenta							1	–17.5		12.6		3.7
Sillago japonica							3	–17.7	0.1	13.3	0.4	3.9
Thryssa hamiltonii	3	–18.5	0.3	12.8	0.2	3.8						
Trichiurus lepturus	1	–19.4		12.1		3.6						
Zoarces gilli	3	–18.1	0.3	10.5	0.4	3.1	3	–19.2	0.2	10.8	0.2	3.1

Supplementary Table S3 δ^{13}C, δ^{15}N, and trophic positions (TP) of fish assemblages collected in the eastern regions of the Yellow Sea (site A and site B) in August 2023. Data represent mean ± 1SD.

Species name	St. A						St. B					
	n	δ^{13}C		δ^{15}N		TP	n	δ^{13}C		δ^{15}N		TP
		Mean	S.D.	Mean	S.D.			Mean	S.D.	Mean	S.D.	
Acropoma japonicum	3	–20.1	0.9	10.9	0.4	3.1						
Benthosema pterotum	3	–21.4	0.5	10.5	0.3	3.0						
Chelidonichthys spinosus	2	–18.3	0.6	10.8	0.7	3.1	2	–16.1	0.7	10.9	0.1	3.1
Conger myriaster	3	–20.1	0.4	12.8	0.7	3.7	7	–19.1	1.4	12.7	1.3	3.7
Dipturus kwangtungensis	2	–17.3	0.2	12.0	0.2	3.5						
Engraulis japonicus	6	–19.5	0.6	9.4	0.9	2.7						
Eopsetta grigorjewi	2	–18.9	0.8	11.8	0.6	3.4						
Euprymna morsei	1	–22.0		8.5		2.5						
Gadus macrocephalus	3	–20.4	0.2	11.1	0.5	3.2						
Hemitripterus villosus	2	–18.0	0.1	13.0	0.2	3.8						
Hexagrammos otakii	1	–18.7		12.1		3.5						
Larimichthys polyactis	5	–20.0	0.4	11.1	0.3	3.2	3	–19.9	1.0	10.1	0.2	2.9
Liparis tanakai	5	–18.9	0.5	11.5	0.5	3.3	4	–18.9	0.7	10.6	0.2	3.1
Lophius litulon	3	–18.3	0.2	11.5	0.4	3.3	3	–18.5	0.5	11.8	0.1	3.4
Pleuronichthys cornutus							2	–18.2	0.5	12.3	0.2	3.5
Raja pulchra	1	–17.7	0.2	11.3	0.1	3.3						
Scomber japonicus	3	–21.3	0.7	10.1	0.3	2.9	9	–19.4	1.5	10.5	0.6	3.0
Sebastes schlegelii	2	–19.3	1.4	11.4	2.4	3.3						
Tanakius kitaharai							1	–18.7		10.3		2.9
Todarodes pacificus							3	–18.9	0.7	11.6	0.6	3.3
Trachurus japonicus	1	–19.6		10.9		3.1	3	–21.1	1.4	11.5	0.5	3.3
Trichiurus lepturus	6	–21.3	0.9	11.0	1.0	3.2	9	–17.1	1.0	11.6	0.5	3.4
Zoarces gilli	4	–19.3	0.4	10.9	0.1	3.2	3	–19.0	0.0	10.6	0.3	3.0

Supplementary Table S4 δ^{13}C, δ^{15}N, and trophic positions (TP) of fish assemblages collected in the eastern regions of the Yellow Sea (site A and site B) in November 2023. Data represent mean ± 1SD.

Species name	St. A						St. B					
	n	δ^{13}C		δ^{15}N		TP	n	δ^{13}C		δ^{15}N		TP
		Mean	S.D.	Mean	S.D.			Mean	S.D.	Mean	S.D.	
Acropoma japonicum	1	–18.8		11.4		3.2						
Apogon lineatus	3	–18.4	0.2	10.8	0.5	3.0						
Argyrosomus argentatus	3	–17.9	0.4	13.0	0.6	3.7	3	–18.0	1.4	12.9	0.3	3.7
Caelorinchus multispinulosus							1	–19.4		11.8		3.3
Chelidonichthys spinosus	3	–17.7	1.3	11.0	0.5	3.1	3	–17.3	1.2	10.9	0.5	3.1
Collichthys lucidus							3	–21.2	0.5	10.3	0.2	2.9
Collichthys niveatus	3	–18.4	0.9	10.2	0.4	2.9						
Conger myriaster	3	–20.3	0.7	11.9	0.4	3.4	3	–19.3	1.1	12.9	0.2	3.7
Cottiusculus schmidti	3	–18.5	0.1	11.0	0.0	3.1						
Engraulis japonicus	3	–19.2	1.2	9.9	1.0	2.8						
Eopsetta grigorjewi	3	–18.7	0.2	11.4	0.2	3.2						
Erisphex pottii	3	–18.8	0.9	9.6	0.4	2.7						
Euprymna morsei	3	–21.4	0.2	9.7	0.1	2.7						
Hemitripterus villosus	2	–18.6	0.1	12.1	0.4	3.4	1	–18.6		12.6		3.6
Hexagrammos otakii	3	–19.7	0.8	11.0	0.4	3.1						
Johnius belengerii	3	–17.6	1.0	12.9	0.2	3.7						
Larimichthys polyactis	3	–19.4	0.3	10.9	0.8	3.1	3	–19.6	0.3	10.6	0.6	3.0
Liparis tanakai	3	–18.3	0.2	11.3	0.2	3.2						
Loligo japonica	3	–17.1	0.2	11.8	0.4	3.3						
Lophius litulon	3	–18.1	0.4	12.3	0.2	3.5	3	–17.0	0.3	12.3	0.0	3.5
Nibea albiflora	1	–18.9		12.6		3.6						
Okamejei kenojei	3	–17.6	0.7	12.3	1.2	3.5	1	–17.9		12.4		3.5
Pagrus major	3	–17.0	0.4	12.6	0.9	3.6						
Pampus echinogaster	2	–18.5	0.0	12.3	0.0	3.5	3	–19.6	2.5	10.1	0.6	2.8
Psenopsis anomala						3.1	1	–19.3		11.2		
Scomber japonicus	4	–18.9	0.2	10.6	0.4	3.0	6	–19.9	1.1	10.5	0.5	2.9
Scyliorhinus torazame							1	–17.4		12.0		3.4
Setipinna tenuifilis	3	–18.2	1.5	12.2	0.6	3.5	1	–19.0		11.8		3.3
Sillago japonica	1	–17.7		12.4		3.5	3	–17.6	0.2	13.2	0.4	3.7
Sphyraena pinguis							1	–19.5		13.0		3.7
Tanakius kitaharai							1	–18.9		12.7		3.6
Todarodes pacificus	1	–16.9		11.9		3.4						
Trachurus japonicus	3	–18.6	1.8	11.3	0.3	3.2	3	–18.5	2.1	11.7	0.5	3.3
Trichiurus lepturus	4	–19.2	1.5	11.3	0.9	3.2	1	–20.4		11.3		3.2
Zoarces gilli	3	–18.7	0.8	10.9	0.6	3.1	1	–18.1		10.5		3.0
